# *"...they should be offering it": *a qualitative study to investigate young peoples' attitudes towards chlamydia screening in GP surgeries

**DOI:** 10.1186/1471-2458-10-616

**Published:** 2010-10-18

**Authors:** Angela H Hogan, Rebecca S Howell-Jones, Elizabeth Pottinger, Louise M Wallace, Cliodna AM McNulty

**Affiliations:** 1 Health Protection Agency, Primary Care Unit, Gloucester Royal Hospital, Gloucester, UK; 2 Microbiology and Epidemiology of STIs and HIV (MESH) Department, Health Protection Agency Centre for Infections, London, UK; 3 Applied Research Centre Health & Lifestyles Interventions, Coventry University, Coventry, UK

## Abstract

**Background:**

Despite the known health and healthcare costs of untreated chlamydia infection and the efforts of the National Chlamydia Screening Programme (NCSP) to control chlamydia through early detection and treatment of asymptomatic infection, the rates of screening are well below the 2010-2011 target rate of 35%. General Practitioner (GP) surgeries are a key venue within the NCSP however; previous studies indicate that GP surgery staff are concerned that they may offend their patients by offering a screen. This study aimed to identify the attitudes to, and preferences for, chlamydia screening in 15-24 year old men and women attending GP surgeries (the target group).

**Methods:**

We undertook 36 interviews in six surgeries of differing screening rates. Our participants were 15-24 year olds attending a consultation with a staff member. Data were analysed thematically.

**Results:**

GP surgeries are acceptable to young people as a venue for opportunistic chlamydia screening and furthermore they think it is the duty of GP surgery staff to offer it. They felt strongly that it is important for surgery staff to have a non-judgemental attitude and they did not want to be singled out as 'needing' a chlamydia screen. Furthermore, our sample reported a strong preference for being offered a screen by staff and providing the sample immediately at the surgery rather than taking home a testing kit. The positive attitude and subjective norms demonstrated by interviewees suggest that young peoples' behaviour would be to accept a screen if it was offered to them.

**Conclusion:**

Young people attending GP surgeries have a positive attitude towards chlamydia screening and given the right environment are likely to take up the offer in this setting. The right environment involves normalising screening by offering a chlamydia screen to all 15-24 year olds at every interaction with staff, offering screening with a non-judgemental attitude and minimising barriers to screening such as embarrassment. The GP surgery is the ideal place to screen young people for chlamydia as it is not a threatening place for them and our study has shown that they think it is the normal place to go to discuss health matters.

## Background

*Chlamydia trachomatis *(chlamydia) infection is the most commonly reported sexually transmitted infection in the United Kingdom and 68% of cases occur in people aged 15-24 years [1]. It is largely asymptomatic and untreated infections can have serious public health impacts as the sequelae include pelvic inflammatory disease and ectopic pregnancy [2]. However, it can be easily detected using a Nucleic Acid Amplification Test (NAAT), on either a urine sample or a self taken vaginal swab, and it is easily treated with antibiotics. In 2003 the National Chlamydia Screening Programme (NCSP) was introduced to offer free chlamydia screening to 15-24 year olds (the target group), thereby preventing the development of sequelae and reducing onward transmission.

There were 7,136,000 young people aged 15-24 in the UK in 2007 [3]. Whilst 16% had a chlamydia screen but only 164,538 (2.3%) were screened in a General Practitioner (GP) surgery [4]. In the chlamydia screening pilot studies nearly half of the cases of infection in women were identified in GP surgeries [5]. As 60-70% of young people in the target age group visit the surgery at least annually, the number taking up chlamydia screening could be much higher [5,6].

Previous studies reporting on the attitude and opinions of staff towards chlamydia screening at GP surgeries, and the barriers to offering opportunistic screens [7-9], have found that staff in low screening surgeries reported they were reluctant to raise the topic of chlamydia screening as they thought it may cause offence, particularly if the young person had not attended the surgery for a sexual health consultation [10]. Therefore, in this study, through interviews with the target group we aimed to:

• Determine young people's opinion of being offered a chlamydia screen at their GP surgery and to determine whether these differ in GP surgeries with high and low screening rates.

• Identify what provisions are needed within GP surgeries to optimise the quality and effectiveness of delivery of the NCSP.

## Methods

In 2007-2008, we conducted 36 semi-structured interviews with young people attending six NCSP registered GP surgeries in London, Wirral and Middlesex (three high screening GP surgeries (screening over 3% of target group) and three low screening GP surgeries (screening up to and equal to 3% of target group).

Interviews were chosen as our method of data collection as we wanted to ask open-ended questions, be able to explore opinions about the screening programme, and also to follow up comments to ensure there was no misunderstanding of either the question or response.

### Ethics

Ethical approval for the study was received from Scotland A Research Ethics Committee. Reference 04/MRE10/41. All participants received a £10 gift voucher to compensate them for their time.

### Surgery recruitment

We purposively chose GP surgeries that had previously participated in our focus-group study of healthcare professionals' attitudes to chlamydia screening [10]. We chose to use these surgeries again as they included urban and rural areas and populations; different levels of deprivation score; divergent ethnicity and differing screening rates. Surgeries with a range of screening rates, based on NCSP data, were selected and approached by letter and telephone to the Surgery Manager and consent was obtained from the surgery for us to approach their patients.

### Patient recruitment

A member of the research team recruited and interviewed male and female patients, aged 15-24 years in the GP surgery. This group were chosen as they are the target group to be opportunistically offered chlamydia screening in GP surgeries. Most participants were approached immediately after they had completed their consultation (irrespective of the reason for their consultation). Where it was not going to be possible to approach them following their consultation (due to surgery layout) patients were invited to participate and interviewed prior to their consultation. The presence of the researcher was not highlighted to clinical surgery staff to limit the potential for influencing behaviour (i.e. offering a chlamydia screen within the consultation). The interviews were undertaken at the GP surgery in a private area away from general thoroughfare by RHJ and EF.

All participants received an information leaflet, were asked to give written informed consent and were assured of anonymity and confidentiality. Following the interview they were offered a chlamydia screening pack and they could then, either submit a specimen for testing that day or take it away to return the sample later. The researchers were neither surgery staff, nor involved in the NCSP and were trained in qualitative interview techniques.

### Interview questions

The interview questions were developed, based on previous research with chlamydia screening co-ordinators and GP surgery staff [7,9,10] and informed by constructs of the Theory of Planned Behaviour (TPB) [11].

The Theory of Planned Behaviour (TPB) is an extensively used psychological model for understanding human behaviour [12]. It infers that people are far more likely to behave in a specific way if they form a conscious intention to do so and this intention is the major determinant of whether a behaviour will happen. The model further concludes that the formulation of this intention is derived from the combination of three key factors, each consisting of two further elements:

Personal attitude (whether a person is in favour of doing it) is influenced by:

• a person's belief in the benefit of the outcome (outcome beliefs) and also by

• any reward that they will receive by performing the action (rewards of action)

Subjective norms (how much a person feels social pressure to do it) is influenced by:

• whether they feel others think they should be doing it (normative beliefs)

• and their willingness to do this (motivation to comply)

**Figure 1:**
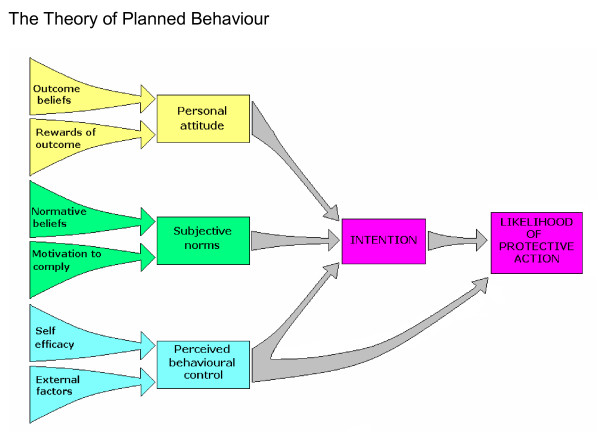
**The Theory of Planned Behaviour**. A diagram to show how the factors of the Theory of Planned Behaviour interact with each other.

Perceived behavioural control (PBC: whether the person feels in control of the action in question) is influenced by:

• the belief that one is capable of performing the action (self-efficacy)

• and barriers/facilitators they feel are beyond their control (external factors) (Figure 1)

Exploring opportunistic screening within a socio-cognitive model provides a strong framework for measure development and analysis. The TPB has been shown to predict variations in intention and behaviour in a wide variety of health activities ranging from contraceptive use to exercise uptake, and thus is a feasible theory to apply to a topic not previously explored in terms of socio-cognitive factors. This study illustrates an initial application of this theory to opportunistic screening, providing identification of the relevant social and cognitive factors involved in opportunistic screening.

Interviews were semi-structured and followed broad topic areas within a TPB framework but encouraged respondents to discuss their perceptions and experiences freely. The broad areas to be discussed included: issues relating to the interviewees' motivation for screening (attitudes), perceived staff and friends' attitudes (subjective norms), perceived barriers to screening and service access both generally and in the GP surgery (PBC), and more general issues such as surgery ambiance, layout, setting and their views on the advantages of using their surgery rather than other sexual health services. In addition, a number of categorical questions were asked to identify participant's previous exposure to the NCSP and to determine the intention of participants screening behaviour. The acceptability and feasibility of potential strategies to increase chlamydia screening in surgeries that had been raised in our previous research with healthcare professionals were explored, including having kits available in the reception area/toilet to take home. Participants were asked to identify factors that may make it easier for a young person to have a chlamydia screen at a GP surgery. In our previous research GP surgery staff were very positive about chlamydia screening kits being available in the reception or the toilets [11]. However, whilst surgeries which use this method of screening have a high turnover of kits, the return rate nationally is low [13]. To understand the barriers preventing young people returning the kits, the participants were asked their opinions on the low return rate. Participants were not asked about their sexual activity or sexual health. The interview schedule was developed to be used as a guide and the respondents were allowed to lead the interviews. The interviews took 20-40 minutes depending on their level of engagement, some of our respondents answers covered several questions at once and the interview was adjusted accordingly. Additional file 1

### Data analysis

All interviews were audio-recorded and transcribed verbatim, then read and checked for accuracy by the qualitative researcher. Data were analysed using Thematic Analysis [14]. After identifying themes, the coding frame was determined using all of the data. The analysis was undertaken by researchers independent of the interviewers. All text was read and re-read before identifying initial themes, noting common themes, and documenting both insights and unforeseen topics. Themes were refined as redundant or infrequent codes were removed or recoded. The themes were then examined in relation to the central topic of concern: the influences on the motivation and behaviour of young people to be screened. We also examined the differing responses of individuals by gender, age, previous experience of screening and whether the surgery had high or low screening rates. In this way we moved from initial to focussed codes. The lead researcher's coding was checked by a second researcher who independently coded four transcripts and any disagreements were resolved by discussion. The agreement was high so no further checks were deemed necessary.

## Results

Of 51 patients invited to participate, 17 declined due to practical reasons (16 for time related factors and just 1 due to sexual health nature of interview) and 36 people agreed. Nine were male and the sample had an age range 15-24 years (mean age 21 years). Of our participants 24 (65%) had never had a chlamydia screen. They were recruited from six GP surgeries with screening rates ranging from 3% to 15% (screening data from 2007 NCSP data). No difference was found in the attitudes and opinions between male and female participants or participants of different age. Neither between people who had had a chlamydia screen previously and those who had not nor between participants from high and low screening surgeries (data not shown), and therefore all results are presented together.

### Intention of participants to accepting a chlamydia screen

We offered a chlamydia screening pack to all people who agreed to be interviewed, which they could then submit whilst still at the surgery or take home and return later. All but one of our participants took a screening kit but due to the confidentiality of our participants we are unable to determine who submitted a specimen following our interviews. However, of our 36 participants 32 reported that they would have been happy to accept a chlamydia screen during their consultation that day. Of these, only one said that being offered a screen in the surgery would have made them anxious. (The four participants who said that they would have declined the screen that day explained that this was because either they were not sexually active or it wasn't convenient at that time to stay for a screen.)

### Personal attitudes to being offered a chlamydia screen

The majority of male and female participants had positive personal attitudes towards chlamydia screening in GP surgeries:

"I'd prefer it at the doctors.... I've been coming here basically since I was born.... so I like coming here"

Interview 2: London, Male 19 yrs

They reported that being offered a screen was easier than having to ask for one and being offered a screen gave patients the opportunity to ask questions:

*"If someone offers *[it to] *me, it kind of makes it easier"*

Interview 14: London, Female 23 yrs

Nearly all participants expressed positive views about being offered a screen during a family planning consultation. However, opinion regarding being offered a chlamydia screen in an unrelated consultation was more diverse. Most participants were either positive:

*"It wouldn't bother me if I got offered it *(in an unrelated consultation) *because if I needed it I could just say yes"*

Interview 4: London, Female 20 yrs

or stated that initially they would be shocked but would still take up the offer:

"I'd be slightly surprised but, um, I guess it would be sensible you know, I would say fair enough, ...they should be offering it."

Interview 6: London, Female, 24 yrs

Four participants expressed negative views about being offered a screen in a consultation unrelated to sexual health but only two participants expressed that they would feel offended if offered a screen.

With regard to receptionist involvement in chlamydia screening, 26 (72%) participants reported that they would accept if a receptionist gave them a leaflet about chlamydia screening, provided it was given in a discreet and non-judgemental way:

"That would be okay, yeah, if they said to you 'would you read that while you're waiting'"

Interview 15: London, Male, 20 yrs

"as long as they didn't shout it out in front of everyone"

Interview 36: Wirral, Female, 17 yrs

Participants' attitudes towards being offered a chlamydia screen by a receptionist were mixed. 11 of the above 26 participants would also accept a screening kit if offered by a receptionist. (2 were not asked due to change of subject) Those that were not happy to accept a kit felt that it was not the role of receptionists to offer screens as they were not health professionals and they were concerned that reception is too open and lacked the privacy to discuss personal matters:

"I would feel little bit, embarrassed if people are around, I'd rather, I don't know it's a bit too open I think"

Interview 35: Wirral, Female, 24 yrs

A large proportion, 26 (72%) of our participants said they would take a home testing screening kit, although some highlighted the importance of the location and discreetness of where the kits actually were situated:

"I think the whole idea of taking it home and doing it yourself it feels straightforward as well and it's convenience, sort of comfortable because you're doing it"

Interview 18: London, Female, 23 yrs

Despite most of the participants expressing positive views about the home testing kits, the majority of the sample actually reported a preference for taking the sample at the surgery rather than doing it at home. The main reason for this was so they would not have to worry about returning the sample:

"yeah, do it while you are here, why not, because it they're taking it home you're not going to bring it back"

Interview 14: London, Female, 24 yrs

### Subjective Norms to being offered a screen at the GP surgery

About half of the participants believed that doctors and nurses did want them to be screened, mostly because it was good for their health:

"yeah I should imagine they'd want you to wouldn't they because they're doctors and they want to make sure you're healthy, you're on top of your health, yeah, I should imagine so yeah"

Interview 35: Wirral, Female, 24 yrs

However, the other participants either felt that doctors and nurses didn't want them to be screened or they were unsure as they had never discussed chlamydia screening with a health professional in their GP surgery:

"I don't know, I don't have any indication that they do"

Interview 13: London, Female, 20 yrs

Participants reported much more positive views regarding their family, partner and friends' beliefs about them getting screened. Twenty-five participants believed that they would either be positive about it or that it would not bother them:

"My friends and family wouldn't think any less of me; in fact I think they'd think more of me for going to the doctors and getting checked"

Interview 4: London, Female, 20 yrs

Only a minority of participants said that they would not tell their friends, partner and family and one person explained that their friends would laugh about it:

*"They *(my family) *would be fine with it......my friends would probably laugh"*

Interview 31: Wirral, Male, 20 yrs

### Perceived Behavioural Control

#### Barriers to accepting a chlamydia screen at the surgery

To understand the barriers which may prevent young people from having a chlamydia screen at their GP surgery or taking a home testing kit, participants were asked what factors they thought would make it more difficult for a young person (either themselves or others) to have a screen at the surgery. The main themes are explored below.

##### Embarrassment

The most common theme that emerged was related to embarrassment. Nineteen participants believed that some young people would feel too embarrassed to accept a screen from surgery staff and to admit that they had practised unprotected sex:

*"They don't want to embarrass themselves by coming and asking and stuff*"

Interview 33: Wirral, Female, Age Unknown

Similarly, when discussing returning a home testing kit taken from the surgery embarrassment remained a barrier in case they were seen by someone they knew:

"You don't want anyone to see you coming back in with it, I think people feel a little bit embarrassed"

Interview 18: London, Female, 23 yrs

Another barrier linked to embarrassment is fear of being judged by the staff at the surgery and fear of parents finding out:

"Even though obviously doctors are you know confidentiality is important I probably predict some people will be scared in case they said to their mum"

Interview 32: Wirral, Female, 18 yrs

##### Scared of results and outcome

The next most common barrier suggested by participants (regarding either being screened in the surgery or taking a home testing kit) was that other young people may be scared of the results or the outcome of the results, e.g. getting a positive result and having to tell previous partners. This was mentioned 16 times by participants when discussing being screened at the surgery:

"Worried about what the outcome could be and stuff, some people rather run away from it than face it"

Interview 23: Middlesex, Female, 17 yrs

And 14 times when discussing home testing kits:

*"Maybe they're scared of the results and therefore they don't want to continue with it" *(returning a home testing kit)

Interview 31: Wirral, Male, 20 yrs

##### Lack of knowledge

Participants identified lack of knowledge as another barrier, including knowledge about chlamydia, its sequelae, and the screening process:

"It's not very clear that all you have to do is pee in a pot"

Interview 24: Middlesex, Male, 20 yrs

*"I think it's just general knowledge they don't really understand how bad it is do they*"

Interview 33: Wirral, Female, Unknown age

It was speculated by participants that some young people would either prefer to ignore the issue which is easy to do when there are no symptoms or they think that it is not important or relevant to them, which prevents them from being screened:

"they think oh go away I don't want to think about that"

Interview 5: Middlesex, Male, 22 yrs

##### Practical barriers

Other practical barriers were also highlighted through the interviews, such as time constraints in consultations for screens in the surgery and forgetting to return the sample for home testing kits:

"I don't think they [doctors and nurses] give you enough time to talk about anything, I feel quite rushed"

Interview 13: London, Female, 20 yrs

"Maybe forgot to get round to it, maybe, you know, sometimes you can have the best intention"

Interview 17: London, Female, 20 yrs

Having specific sessions just for young people was identified as another practical barrier as many reported that they would be worried about seeing people they knew there and also it could be more embarrassing as people would know why they were there:

"I think it has a few drawbacks because in fact specific times aren't convenient for everybody. You come into the doctors and everybody knows at this point, it's when they {are} screening for chlamydia, you don't want anyone to see you going in about this time and that can be a bit embarrassing for some people"

Interview 29: Middlesex, Male, 24 yrs

#### Facilitators for accepting a chlamydia screen at the GP surgery

Participants were also asked to identify factors that may make it easier for other young people to have a chlamydia screen at a GP surgery or to take a home testing kit.

##### Raising awareness

The main factor identified to facilitate more young people to be screened was that of raising awareness and providing more information about chlamydia. It was suggested that this information could be given through marketing campaigns, such as posters, through letters/leaflets in the post and the doctors and nurses talking to the patients about screening:

"Especially if they [doctors] talked about it and offered a test"

Interview 18: London, Female, 23 yrs

"mail it to you and like explaining it to you, what's the risk and how you get it and stuff maybe or like probably posters so you can read it and stuff"

Interview 1: London, Female 20 yrs

##### Characteristic of doctor or nurse

There were six instances in the interviews where patients identified a characteristic of the doctor or nurse which made it easier to accept a screen however, the preferences of these characteristics (such as gender or age) differed between participants. Also, in contrast to the barrier of embarrassment identified, some of the patients reported that they would feel more comfortable with a doctor who they knew. This highlights the importance of offering the patients options of which doctor or nurse they see:

*"Personally *(I) *asked to see a female doctor, it did help the fact that she was younger as well because I think they..., maybe you'd like to think that they can associate a bit better really"*

Interview 30: Female 20 yrs

"I think it would be better if they were older because they would have more experience"

Interview 8: Female 17 yrs

Some participants identified specific characteristics of the doctor or nurse which made it easier to have a screen; the most essential characteristic linking back to the barrier of embarrassment was that of being non-judgemental.

"afraid that the doctors and nurses are gonna judge them, that, that's what I think"

Interview 35: Wirral, Female, 24 yrs

## Discussion

### Main findings

This study has demonstrated that the target group of the NCSP attending GP surgeries consider surgeries to be an acceptable venue for opportunistic chlamydia screening and furthermore, they think it is the responsibility of General Practitioners and Practice Nurses to offer screening. Participants in this study felt strongly that surgery staff (including reception staff) should have a non-judgemental attitude and that they did not want to be singled out as 'needing' a chlamydia screen. Interviewees reported a strong preference for providing the sample at the surgery rather than taking a home testing kit to return later. This may go some way to explain the discrepancy that although many of our participants thought self-testing kits were beneficial, nationally not many are returned once taken from GP surgeries. Only one of our 36 interviewees attending a routine appointment reported that being offered a chlamydia screen in a GP surgery setting would make them anxious, and only a few others would decline the offer, stating that they were not sexually active or lacked time. The positive attitude and subjective norms demonstrated by interviewees suggest that young people would intend to accept a screen if it was offered to them, but that that perceived and practical barriers may mediate the translation of this intention into behaviour. In order to increase the likelihood of behaviour, barriers to accepting a chlamydia screen must be reduced (i.e. removing judgemental attitudes and making specimens easy to provide and hand in at the surgery). Additional file 2

### Strengths

It is a strength of this study that participants were recruited within surgeries, in most cases after they had presented for a consultation for another reason. This therefore replicates the NCSP recommendation of opportunistic chlamydia screening to the target age group. The interview questions for this study were underpinned from the outset by a theoretical model for behaviour as well as being informed by our previous research in this area, providing us with an a-priori, evidenced framework on which the data was explored.

### Weaknesses

We undertook 36 interviews in three geographical areas but recognise that our findings may not be representative of all young people in the UK. However, the surgeries in our study were purposively selected to include urban and rural areas and populations; different levels of deprivation score; divergent ethnicity and differing screening rates. Additionally, whilst the TPB was a beneficial component of the study, it was beyond the parameters of this study to assess the relative influence of each dimension. Further quantitative work could identify the most salient influences on behaviour and determine how these could be targeted to increase uptake of screening.

### Other work in this area

Whilst other qualitative studies exploring attitudes of the target group to chlamydia screening have been performed, these have recruited participants from education, sexual health or sports establishments or have involved participants who have either already been offered a chlamydia screen or do not live in the UK and therefore have no knowledge of the NCSP [15-19]. We recruited participants in GP surgeries and were able to discuss their acceptance of opportunistic chlamydia screening at their surgery. Our previous research in GP surgeries, exploring the staff opinions towards opportunistic chlamydia screening, found a strong association between staff opinions and the number of chlamydia screens performed [7]. In this study however, the opinions of our participants recruited in high and low screening surgeries were similar suggesting, that the opinions of staff do not adversely affect their patients' attitudes towards, or opinions of, chlamydia screening and that patient attitudes are probably not responsible for differences in screening rates as suggested by some GP surgery staff [9].

Recent studies have found that young people feel the need to normalise screening and our study affirms these findings [20,21]. It has also been previously reported that young people want screening to be based on age criteria rather than need, stating that if asked they would lie about their sexual history [20,22]. Therefore, we propose that chlamydia screening should be offered to all 15-24 year olds without first ascertaining any sexual history. Offering a test to *all *of the target group would remove the problem of young people feeling 'singled out'.

Another study found that whilst men appreciate the convenience of screening in non-medical settings, women found the public nature of these settings inhibited them from taking part [19]. Our study demonstrated that young people of both gender attending GP surgeries would be happier being screened at their surgery rather than anywhere else. Studies have now shown that men visit their surgery as often as women [23] and that prevalence is as high in men as in women [24]. Men should, therefore also be offered chlamydia screening at their GP surgery.

When comparing venues and considering levels of positivity it is worthy of note that for sustainability, in 2008-2009 general GP surgeries returned the highest levels of positive results (6.5%) when compared to the most popular opportunistic venue 'education settings' which provide 3.4% positivity (excluding all sexual health settings) [13]. Education settings are excellent for raising the profile of the NCSP but they require consistent input from the chlamydia screening co-ordinators to continue to screen the numbers of young people required to meet the annual target as education setting populations change regularly. Consequently, it is a key future objective of the NCSP is to enlist greater support from GP surgeries [25].

Several studies, carried out with people in sexual health establishments or with people newly diagnosed with positive chlamydia infections have reported stigma as a problem, both when attending sexual health services and when receiving the diagnoses [26,27]. Further negative factors included guilt, embarrassment linked to treatment delays and poor outcome [15,27]. Screening in the GP surgeries (as opposed to sexual health settings) and providing the specimen immediately in the surgery was seen by our participants as a way to minimise any embarrassment. Our study has shown that not only do young people not object to being opportunistically offered a chlamydia screen, they think that surgery staff should be doing this.

Previous work has reported that young people felt more education and information were needed surrounding chlamydia [27], and our study suggests that information about how a screen is collected might also increase uptake. Recent statistics show that education surrounding chlamydia has been effective in England; in 2008 the Office for National Statistics reported that 91% of people aged 16-24 years knew that chlamydia is a sexually transmitted infection and 70% knew it does not always cause symptoms [28]. However, information needs to be delivered continually and to aid this, the NHS launched a campaign aimed at young people called 'Worth Talking About' in January 2010. It has been devised to encourage open and honest discussion between young people, their friends and their families to build a culture that frames sexual behaviour among young people as a normal part of their development. The campaign covers five areas, one of which is chlamydia testing and a TV advert was released in January 2010 to talk openly about chlamydia and to accept a screen if they are offered one [29]. Such campaigns should help with our finding that chlamydia screening needs be normalised.

## Conclusions

Our study suggests that, given the right environment, young people attending GP surgeries will accept chlamydia screening when offered.

Primary Care Trusts (PCTs) and GP surgeries have a role in ensuring the right environment for chlamydia screening in surgeries is achieved. This includes raising the profile of GP surgery based screening and encouraging, with the provision of training when necessary, GP surgery staff to offer chlamydia screens more widely. PCTs should raise awareness of chlamydia screening to GPs helping them to determine how they can achieve the Vital Signs Indicator target for 2010-2011 of screening 35% of young people registered at their surgery at least annually. Finally, PCTs should ensure GP surgery staff are aware that young people prefer to be screened at the surgery in preference to other venues.

Within GP surgeries it is important that staff are aware that 15-24 year olds would prefer to be screened at the surgery rather than other settings and that they are unlikely to be offended by an offer of a chlamydia screen. They would prefer to be offered a chlamydia screen rather than having to ask for one, and they would prefer to provide the sample immediately rather than taking a self collection chlamydia kit home. It should be stressed that all staff in the surgery should display a non-judgemental attitude when discussing chlamydia screening and that it should be offered to all young people in the target group at every opportunity to prevent them feeling singled out. All staff can have a role to improve screening rates and our study found that young people are not offended by being approached by any member of the team, provided there is privacy. If surgeries provide home testing kits it is important that the return process is made as discreet as possible to maximise return rates. Additional file 3

## Competing interests

The authors declare that they have no competing interests. The Project is funded by the Funding was provided by the Health Protection Agency.

## Contributions of each author

CMcN and RHJ were responsible for the conception and design of the study, RHJ and AH were responsible for the collection and assembly of data, EP, LW and AH were responsible for the analysis and interpretation of data, AH drafted the article and all authors critically reviewed the article prior to submission.

## Pre-publication history

The pre-publication history for this paper can be accessed here:

http://www.biomedcentral.com/1471-2458/10/616/prepub

## Supplementary Material

Additional file 1 **Interview Schedule**. The full list of proposed subject topics and questions used for the semi-structured interviews Click here for file

Additional file 2 **Summary of Findings**. A diagram to show how our findings are interpreted using the Theory of Planned Behaviour Click here for file

Additional file 3 **Implications for Stakeholders**. A diagram to show how our findings can be translated to aid GP surgeries to undertake more chlamydia screens Click here for file
