# Supplementary material for: "...they should be offering it": a qualitative study to investigate young peoples' attitudes towards chlamydia screening in GP surgeries
Source: BMC Public Health. 2010 Oct 18;10:616. doi: 10.1186/1471-2458-10-616 (PMC2965724; doi:10.1186/1471-2458-10-616)
Supplement: Additional file 1 — Interview Schedule. The full list of proposed subject topics and questions used for the semi-structured interviews [file 1471-2458-10-616-S1.DOC]

# Questionnaire for patients aged 15–24years attending practices

1. Chlamydia screening has been running in this area for over a year. What do you understand by the term chlamydia screening?
2. Have you ever been offered a chlamydia screen?

*Prompt: If yes, when did you have your last screen?*

1. What would you expect to be in a chlamydia screening kit?
2. Explain how you think you would give a sample for chlamydia screening.
3. If you were offered a chlamydia screen at the practice today would you accept it?
4. Where would you prefer to have a chlamydia screen? *Probe: surgery, family planning clinic or other.*
5. Are there any advantages in coming to the surgery rather than going to family Planning or Brook Clinics for a chlamydia screen? (Hayter 2005)
6. What are your views on being offered a chlamydia screen?

*Prompt: Do you think it is relevant to you?*

*If NO: Why not, what would make it more relevant? Probe with men.*

1. Are you of the opinion that the doctors and nurses think you, and other 15–24 year olds, should have a chlamydia screen in this practice?
2. How do you find *(or if not previously offered a test, how do you think you would find)* discussing chlamydia with different members of staff?

*Probe differences in age, gender, ethnicity, doctors, nurses or receptionists approach/ confidence in the staff etc.*

1. What are your views on being offered a chlamydia screen by a nurse?
2. What are your views on being offered a chlamydia screen by a doctor?
3. What are your views on being offered a chlamydia screen by a receptionist?
4. What would your family, partner, or friends think of you having a chlamydia screen in this practice?

*Prompt: discuss further with men about their role in the relationship.*

1. Are there things that would make it difficult for you to have a chlamydia screen in this practice?

*Prompts: possibly meeting people you know at this surgery; having enough time to talk about chlamydia with the doctors or nurses in a consultation; or embarrassment.*

*Follow-up on any points raised.*

1. Are there things that would make it easier for you to have a chlamydia screen in this practice?

*Prompts: specific times when only young people can attend - would you like to have similar options at this surgery? Ease of making an appointment at the practice?*

*Cover timing/location of practice. Are you comfortable: (i) sitting in the waiting room (ii) or at the reception desk? Cover urine vs self-taken vulvo-vaginal swabs.*

1. What do you think are the reasons why young people don’t take up the offer of a chlamydia screen, in this practice?
2. What are your views on being offered a chlamydia screen during a family planning consultation?
3. What are your views on being offered a chlamydia screening test during a consultation that is not for family planning?
4. What would you feel if you came in with a sore throat or any other illness and were asked if you would like a chlamydia screen?
5. In what way is this practice young person friendly?

*Prompt: Clinics? Ask about information displayed in the waiting room, on the* ***posters or leaflets****? Are there any that are appropriate for young people on* ***chlamydia****? What do they think of the chlamydia posters or small cards handed out?*

1. In some surgeries the receptionists give patients leaflets about chlamydia screening, what do you feel about this? *Prompt: about privacy, would they then ask staff for a kit?*
2. Some surgeries leave screening kits in the reception area or waiting room. If this happened in this surgery, do you think you would want to take one? *Prompt: if not, why not?*
3. Many young people take the screening kit from a doctor, nurse or reception area, and do not return a sample – why do you think that is?
4. How can this practice encourage young people to take a screening kit **and** return a sample?
5. Some doctors and nurses ask patients to give a urine sample *(or self taken vaginal swab for women: state which ever sample is required locally)* immediately in the surgery, rather than giving them the kit to take home.
   1. *How would you feel about this?*
   2. *How do you think staff should ask for a sample to increase return?*

1. If the practice wrote to you offering you a chlamydia screen, how would you feel about this? *How likely would you be to visit the surgery for a screening test following receipt of a letter?*
2. How do you think chlamydia screening should be offered in this surgery?
