# Supplementary material for: "...they should be offering it": a qualitative study to investigate young peoples' attitudes towards chlamydia screening in GP surgeries
Source: BMC Public Health. 2010 Oct 18;10:616. doi: 10.1186/1471-2458-10-616 (PMC2965724; doi:10.1186/1471-2458-10-616)
Supplement: Additional file 2 — Summary of Findings. A diagram to show how our findings are interpreted using the Theory of Planned Behaviour [file 1471-2458-10-616-S2.DOC]

**Factors affecting a young persons decision to accept a chlamydia screen if it offered at the GP surgery**.

The **Personal Attitude** of young people accepting a chlamydia screen is influenced by:

Feeling comfortable at the GP surgery and being offered a screen rather than having to ask for one.

Ensuring all surgery staff have a non-judgemental attitude.

Facilitating their preference to do the test at the surgery. rather than taking it home.

A young persons **Subjective Norms** for accepting a chlamydia screen are positively influenced by:

Young people feeling surgery staff want them to be screened for the good of their health.

Thinking their family and friends would approve of them being screened.

Normalising screening by offering it to all 15-24yr olds.

Factors affecting a young persons **Perceived Behavioural Control** towards accepting a chlamydia screen include:

Wanting more information about the possible effects of undiagnosed chlamydia infection.

Being able to choose who they see at the surgery to discuss screening.

Minimising embarrassment by positioning of kits and promoting ease of testing and treatment.

**Increased intention to accept a chlamydia screen if it is offered within the GP surgery.**
