# Supplementary material for: "...they should be offering it": a qualitative study to investigate young peoples' attitudes towards chlamydia screening in GP surgeries
Source: BMC Public Health. 2010 Oct 18;10:616. doi: 10.1186/1471-2458-10-616 (PMC2965724; doi:10.1186/1471-2458-10-616)
Supplement: Additional file 3 — Implications for Stakeholders. A diagram to show how our findings can be translated to aid GP surgeries to undertake more chlamydia screens [file 1471-2458-10-616-S3.DOC]

Implications for chlamydia screening at Primary Care Trusts and practice level

Primary Care Trusts

- Inform primary care staff that young people attending their surgery prefer to be screened in the GP surgery.
- Raise awareness of young people that chlamydia screening is available at GP surgeries.
- Provide training to all primary care staff on communication skills.

GP Surgeries

Increase staff awareness of the following:

- Patients prefer, and therefore should be, offered a screen rather than having to ask for one.
- Chlamydia screening should be normalised by offering all 15-24 year olds screening at every consultation thereby removing any associated stigma.
- Chlamydia screening needs to be offered in a non-judgemental way to maximise uptake.
- Chlamydia screening samples should be collected immediately, rather than taking home self collecting kits for later use at home.
- Any facilities for collection and return of samples outside of a consultation need to be very discreet.
- Receptionists are ideally placed to initiate the notion of chlamydia screening by handing out leaflets or invitation cards.
- If receptionists are situated in a private area, patients do not mind being spoken to by them about chlamydia screening.
